# Multiple spontaneous small bowel perforations due to clozapine—Case report

**DOI:** 10.1016/j.ijscr.2018.10.067

**Published:** 2018-11-01

**Authors:** A. Rodrigues, A. Duarte, A. Marques, M. Magalhães, R. Camarneiro, R. Silva, Á. Ferreira, I. Dionísio, L. Val Flores, M. Brito e Melo

**Affiliations:** a General Surgery Department of Centro Hospitalar do Oeste, Portugal; b Internal Medicine Department of Centro Hospitalar do Oeste, Portugal

**Keywords:** Small bowel perforation, Clozapine, Antipsychotic drugs, Case report

## Abstract

**Introduction:**

Spontaneous free perforation of the small bowel is unusual. There are many causes of spontaneous small bowel free perforation such as: immune-mediated or inflammatory, infections, drugs and biological agents, congenital, metabolic, vascular and neoplasm.

A severe adverse effect of antipsychotic drugs is intestinal ischemia, which could lead to perforation.

**Case presentation:**

The authors report the clinical case of a 42-year-old schizophrenic patient, smoker, medicated with clozapine 600 mg per day, admitted to the emergency room with diffuse abdominal pain. On physical examination the patient presented abdominal rebound tenderness and peritoneal sign with raised inflammation markers and the abdominal tomography revealed pneumoperitoneum.

An emergency laparotomy revealed multiple jejunal and ileal perforations.

The patient was subject to small bowel resection complicated with anastomosis dehiscence, respiratory tract infection and intra-abdominal abscess.

Histologic specimens showed nonspecific inflammatory findings with ischemia.

The main infectious, inflammatory, congenital, auto-immune and vascular causes of spontaneous perforations were excluded.

**Discussion:**

Spontaneous free perforation of the small bowel is uncommon. An intestinal ischemia might be a rare adverse effect of antipsychotics. However, only colon perforations due to ischemic colitis are described in the literature. While diagnosis of the precise cause might be challenging, and after excluding other causes, these perforations may be attributed to an adverse effect of clozapine.

**Conclusion:**

The cause was attributed to clozapine as the other etiologies were excluded. The clozapine’s reintroduction is controversial. In this case the clozapine dose was reduced successfully.

## Highlights

• Spontaneous free perforation of the small bowel is unusual.
• Intestinal perforation is a rare adverse effect of clozapine.
• This complication can affect young patients taking psychotic drugs.
• After perforation, clozapine’s suspension is recommended.
• In special cases, dosage reduction seems to be safe.

## 1 Introduction

Spontaneous free perforation of the small bowel is rare [1]. There are many causes of spontaneous small bowel free perforation such as: immune-mediated or inflammatory, infections (viral, bacteria, parasites and protozoa), drugs and biological agents, congenital, metabolic, vascular and neoplasm [1].

The pre-operative diagnosis is difficult and sometimes it remains challenging postoperatively.

First and second generation antipsychotics commonly cause mild gastrointestinal hypomotility and rarely intestinal ischemia and necrosis. Most cases are related to phenothiazines and more recently several cases have been reported with clozapine, an atypical antipsychotic with antimuscarinic activity [2,3].

The risk of developing ischemic colitis increases when anticholinergic drugs are associated [4].

In this study, a case report of spontaneous small bowel perforations is presented.

This work has been reported in line with the SCARE criteria [5].

## 2 Case presentation

A 42-year old obese, smoker and schizophrenic male was medicated with 600 mg of clozapine per day. He was admitted to the emergency department with a 2 week history of diffuse abdominal pain, abdominal distention, anorexia and semi-liquid stools. On physical examination he had 110/63 mmHg of blood pressure, he was tachycardic (heart rate = 112 beats per minute), febrile (temperature = 38.5 °C) and presented abdominal tenderness and peritoneal sign.

Laboratory investigations showed a hemoglobin of 13.6 g/dl, an increase in inflammatory markers (white blood cells 13.2 × 10^9^/l, C-reactive protein >32 mg/dl) and a renal insufficiency (creatinine 3.02 mg/dl, urea 189 mg/dl).

**Figs. 1 and 2:**
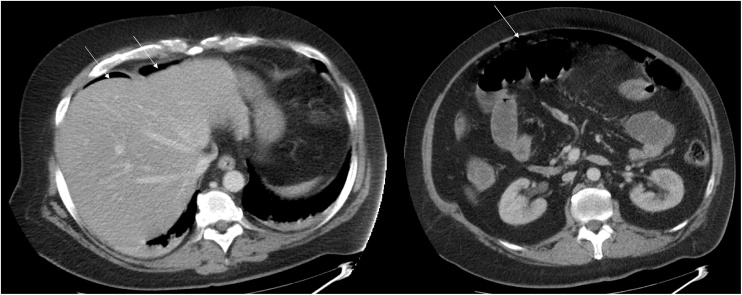
Abdominal computed tomography showing pneumoperitoneum.

An upright abdominal X-ray demonstrated a pneumoperitoneum which was confirmed by the abdominal and pelvic computerized tomography (Figs. 1 and 2 ).

**Figs. 3 and 4:**
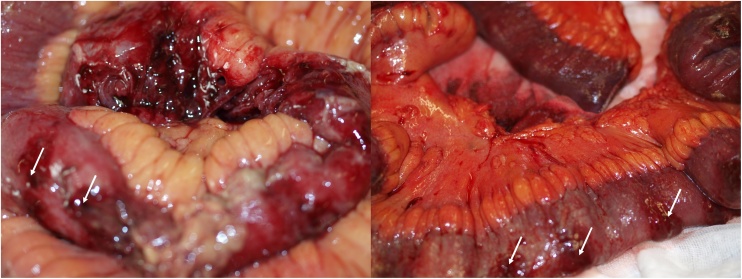
Multiple distal jejunum and ileum perforations (arrow).

He was subjected to an emergency laparotomy where multiple punctiform perforations (holes smaller than 1 cm) in the anti-mesenteric border of the distal jejunum and ileum were identified. Purulent peritonitis was present. A small bowel resection of 1.5 m was done (Figs. 3 and 4 ).

On the second day of the postoperative period, an anastomosis dehiscence was registered. A subsequent re-laparotomy was needed. An anastomosis and caecum resection was done with the creation of an end-ileostomy and a colostomy. During hospitalization he had a respiratory tract infection which was treated with broad-spectrum antibiotics and an intra-abdominal abscess which was treated with percutaneous drainage. On the 28th day of hospitalization the patient was discharged.

Histologic specimens revealed non-specific inflammatory findings with ischemia.

The investigation was carried out with microbiologic cultures, serologic tests, laboratory tests, endoscopic exams with biopsies and other diagnostic exams. The main causes of spontaneous small bowel perforation were excluded, such as, infectious (cytomegalovirus, tuberculosis, bacterial, parasitic and protozoal), immune (Crohn’s disease, celiac or gluten-sensitive enteropathy and vasculitis), congenital (Meckel diverticulum and small bowel diverticulum or duplication), vascular and neoplastic.

The dose of clozapine was reduced because the suspension was not viable and a restoration of the bowel continuity was done ten months later.

## 3 Discussion

The main causes of spontaneous small bowel perforation were excluded, such as, infectious (cytomegalovirus, tuberculosis, bacterial, parasitic and protozoal), immune (Crohn’s disease, celiac or gluten-sensitive enteropathy and vasculitis), congenital (Meckel diverticulum and small bowel diverticulum or duplication), vascular and neoplastic [1].

Some possible causes described in the literature as the cause of multiple small bowel perforations (trauma, typhoid, tuberculosis, amoebiasis, myeloid leukaemia and vasculitis [6]) were also excluded by clinical history and diagnosis complementary exams.

Intestinal ischemia, in particular, ischemic colitis is an uncommon adverse effect of antipsychotic agents, more commonly found with phenothiazine drugs and atypical neuroleptics such as clozapine. Clozapine is the atypical antipsychotic drug most commonly described in the literature as the cause of ischemic colitis [4].

An explanation for induced gastrointestinal necrosis is their antimuscarinic activity, especially when associated with other antipsychotropic drugs [2]. The hypomotility due to clozapine may lead to an increase in intraluminal pressure and subsequent mucosal ischemia and necrosis, which can lead to perforation [2,7].

Another ischemic mechanism could be attributed to the effects of antipsychotics on dopamine. In fact, dopamine could improve the mesenteric perfusion because of its vasodilatory effects at low dosage. The inhibition of dopamine-dependent mesenteric vasodilation, due to the antidopaminergic effect of antipsychotics, could play an additional part in digestive ischemia [8].

Antiserotoninergic properties of clozapine could also explain gastro-intestinal complications. Clozapine antagonize 5HT_2_ and 5HT_3_ receptors which lead to reduced intestinal peristalsis, mucosa secretions, nociception and possibly intestinal sensitivity to distension. At its worst it could result in intestinal perforation [9,10].

Besides clozapine, there are other risk factors described in the literature for intestinal ischemia (HIV + enteritis, overweight, digestive infection and chronic constipation).

The intestinal perforation due to clozapine is rare and only colonic perforations are described in the literature [2,3,11]. This article is the first case report of small bowel perforations.

In this case, there was no association of antipsychotic drugs, besides clozapine, as described in the literature [2]. The only risk factor presented was the obesity.

However, clozapine perse could induce ischemia by antimuscarinic, antidopaminergic and antiserotoninergic activity (described above). This intestinal ischemia caused by these three mechanisms could be in small bowel and could explain the multiple punctiform perforations presented in this case report.

When other medication is not effective, the reintroduction of clozapine after severe adverse drug effects is a clinical dilemma. There are few positive case reports regarding the reintroduction of clozapine after severe gastrointestinal adverse effects. However, after intestinal perforation, there is only one case report described, where the clozapine dose is reduced [3]. Here, it was not possible to withdraw clozapine completely since there was no effective alternative drug. The clozapine dose was reduced.

## 4 Conclusion

Intestinal perforation is a rare adverse effect of clozapine. This complication can affect young patients taking psychotic drugs.

The occurrence of non-specific clinical symptoms such as abdominal pain with vomiting and/or diarrhea should be taken into account. Clozapine’s suspension is recommended, but in special cases, dosage reduction seems to be safe. Howbeit, more studies are needed.

## Conflict of interest

There are no conflicts of interest.

## Sources of funding

This research did not receive any specific grant from funding agencies in the public, commercial, or not-for-profit sectors.

## Ethical approval

This study is exempt from ethical approval in my institution.

## Consent

Written informed consent was obtained from the patient for publication of this case report and accompanying images.

## Author contribution

Ana Rodrigues – care of the patient, study concept and written the article.

António Duarte- responsible surgeon, review article.

Adriano Marques, Mariana Magalhães – care of the patient, review article.

Ágata Ferreira, Isabel Dionísio – participated in surgery, review article.

Rita Camarneiro, Regina Silva, Luis Val-Flores, Margarida Brito e Melo – review article.

## Registration of research studies

In accordance with the Declaration of Helsinki 2013, all research involving human participants has to be registered in a publicly accessible database.  Please enter the name of the registry and the unique identifying number (UIN) of your study.

## Guarantor

Ana Rodrigues.

## Provenance and peer review

Not commissioned, externally peer reviewed.
